# The Roles of β-Tubulin Mutations and Isotype Expression in Acquired Drug Resistance

**Published:** 2007-04-27

**Authors:** J. Torin Huzil, Ke Chen, Lukasz Kurgan, Jack A. Tuszynski

**Affiliations:** 1 Department of Oncology, University of Alberta, Edmonton, Alberta; 2 Department of Computer and Electrical Engineering, University of Alberta, Edmonton, Alberta, Canada

**Keywords:** Tubulin, Microtubule, Isotype, Paclitaxel, Cancer, Resistance, Mutant

## Abstract

The antitumor drug paclitaxel stabilizes microtubules and reduces their dynamicity, promoting mitotic arrest and eventually apoptosis. Upon assembly of the α/β-tubulin heterodimer, GTP becomes bound to both the α and β-tubulin monomers. During microtubule assembly, the GTP bound to β-tubulin is hydrolyzed to GDP, eventually reaching steady-state equilibrium between free tubulin dimers and those polymerized into microtubules. Tubulin-binding drugs such as paclitaxel interact with β-tubulin, resulting in the disruption of this equilibrium. In spite of several crystal structures of tubulin, there is little biochemical insight into the mechanism by which anti-tubulin drugs target microtubules and alter their normal behavior. The mechanism of drug action is further complicated, as the description of altered β-tubulin isotype expression and/or mutations in tubulin genes may lead to drug resistance as has been described in the literature. Because of the relationship between β-tubulin isotype expression and mutations within β-tubulin, both leading to resistance, we examined the properties of altered residues within the taxane, colchicine and *Vinca* binding sites. The amount of data now available, allows us to investigate common patterns that lead to microtubule disruption and may provide a guide to the rational design of novel compounds that can inhibit microtubule dynamics for specific tubulin isotypes or, indeed resistant cell lines. Because of the vast amount of data published to date, we will only provide a broad overview of the mutational results and how these correlate with differences between tubulin isotypes. We also note that clinical studies describe a number of predictive factors for the response to anti-tubulin drugs and attempt to develop an understanding of the features within tubulin that may help explain how they may affect both microtubule assembly and stability.

## Introduction

**Figure 1.**
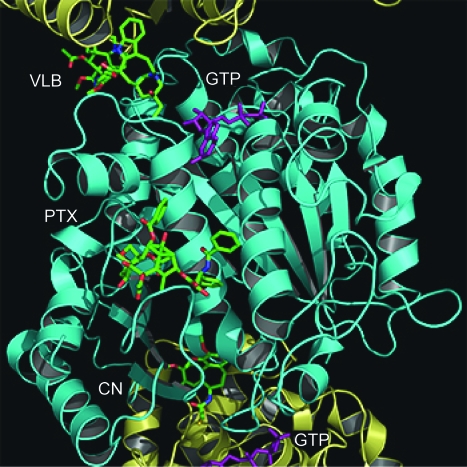
Paclitaxel, colchicine and *Vinca* binding sites on α/β tubulin protofilament. Shown here is a cartoon representation of a protofilament with superimposed drug molecules, shown as space filling spheres. Structures of the paclitaxel (PTX), colchicine (CN) and vinblastine (VLB) from structural files 1JFF, 1SA0 and 1Z2B have been superimposed and fit back onto the 1SA0 structure to obtain the relative positioning of each drug within the protofilament. A single α/β-tubulin heterodimer comprises the β tubulin monomer (cyan) in the center of the frame and two α tubulin monomers (yellow) at the top and bottom of the frame. The GTP at the non-exchangeable and GDP at the exchangeable site are colored purple. **Palitaxel, Colcicine and Vinblastine Binding Sites**

Microtubules (MTs) are large multimeric protein complexes that form hollow cylinders. They are constructed from repeats of a heterodimer of two 55 kDa proteins known as α and β tubulin. The formation of the tubulin dimer from the α/β monomers is essentially irreversible, locking a single molecule of GTP within the non-exchangeable nucleotide binding site of α tubulin (Weisenberg et al. 1976). Following the formation of the tubulin dimer, an additional molecule of GTP binds the β-subunit and subsequent subunit addition then brings the exposed, nucleotide bound β-tubulin into contact with α-tubulin from a separate tubulin dimer (Figure 1). This results in the formation of long chains of tubulin dimers known as protofilaments. This interaction at the α/β tubulin interface, within the growing protofilaments, promotes the hydrolysis of bound GTP, providing the conformational flexibility required during the polymerization-depolymerization cycle (Wang and Nogales, 2005). *In vitro*, these filaments have been shown to associate laterally, forming sheets which are then thought to curl in on themselves to form a complete MT. MTs observed *in vitro* are composed of 11 to 18 individual protofilaments, whereas most microtubules that are observed *in vivo* are made up of only 13 (Unger et al. 1990). MTs are involved in numerous critical, cellular processes including mitosis, cellular motility, maintenance of cellular morphology, and the activity of cell surface receptors. (Pichichero and Avers, 1973; Hyams and Stebbings, 1979). Here we discuss the role of MT dynamics and how tubulin’s involvement at the molecular level may affect processes such as chromosome segregation and cell division. This will be followed by a brief discussion of how this process relates directly to the chemotherapeutic treatment of cancer and the drugs that are used in this process. Finally, a discussion of the development of resistance mechanisms to these drugs and how these relate to tubulin, the expression of isotypes and acquired mutations will hopefully provide insight into the ever-increasing complexity of this system.

### Tubulin structures

Several crystallographic structures of tubulin as it is found within several MT-like conformations are now available from the RCSB Protein Data Bank (PDB) (Nogales et al. 1995; Li et al. 2002; Wang and Nogales, 2005). The first tubulin structure, 1TUB, was crystallized as a flat Zn^2+^ induced sheet of antiparallel protofilament-like end-to-end α/β dimer repeats, using docetaxel as a stabilizing agent (Nogales et al. 1998). Due to difficulties in fitting electron density, this structure contains misalignments and was superseded by 1JFF, in which paclitaxel was utilized as a stabilizing agent (Lowe et al. 2001). Similarly, a third structure 1TVK uses epothilone A, which binds at the same taxane binding site, stabilizing the MTs (Nettles et al. 2004). Additional structures use a stathmin-like domain to produce crystals, the earliest, 1FFX, was derived from the 1TUB data (Gigant et al. 2000). Two higher resolution structures, 1SA0 and 1SA1 followed, producing structures of the colchicine and podophyllotoxin bound complexes (Ravelli et al. 2004), while both the colchicine and vinblastine binding sites are observed in 1Z2B (Gigant et al. 2005). Structurally, α and β tubulin are known to be similar, indistinguishable at a resolution of 6 Å, yet share only 40% amino acid identity (Li et al. 2002). Each tubulin monomer can be divided into three distinct domains. The amino terminal domain (composed of residues 1–205), an intermediate domain (residues 206–381) and a carboxy terminal domain, which contains the flexible C-terminal tail (residues 382–444). Unfortunately, while there is a wealth of information on the structure of tubulin, little is known about the functional significance related to its structural features. This is almost entirely due to the lack of molecular understanding of the dynamic properties of tubulin dimers and how they behave within microtubules.

### Microtubule structure and dynamics and the effect of drug interactions

MTs exhibit an irregular temporal pattern of assembly and disassembly, which has been termed dynamic instability (Mitchison and Kirschner, 1984). The process of dynamic instability features episodes of distinct catastrophes that result in MT collapse, and rescues that lead to microtubule regrowth (Bayley et al. 1990; Drechsel et al. 1992; Odde et al. 1995). The assembly and disassembly processes of MTs, both *in vivo* and *in vitro*, have been extensively studied and are regulated by mechanisms that are sensitive to temperature, pH and ionic concentrations that can accommodate the critical roles played by MTs at different phases of the cell cycle. Following the initial nucleation of a MT, the addition of subsequent tubulin dimers can occur at either of its free ends. Both polymerized and unpolymerized tubulin monomers can bind free GTP, and upon assembly, the energy of hydrolysis from GTP to GDP is imparted into the tubulin subunits, however the outcome of this process and the fate of the energy released is yet unknown. It has been hypothesized that when β-tubulin becomes bound within a MT, the exchange of bound GDP for GTP is blocked, and as a result, the protofilament maintains its structure as long as there is not an exposed β-tubulin at the growing end. Stabilization of the entire MT structure is normally afforded by the binding of a GTP cap at the end of the MT which shields the terminal β tubulin from a conformational change that is then thought to induce its dissociation (Mitchison and Kirschner, 1984). We believe that at least part of the relatively large amount of energy is stored within the MT itself in the form of potential conformational energy. When a critical amount of this potential energy has been exceeded, it is released in the form of a catastrophic collapse of the entire MT structure.

The dynamic properties of MTs are especially critical during mitosis, when a delicate equilibrium of distances and forces is necessary for proper chromosome alignment prior to segregation (Margolis and Wilson, 1981; Horio and Hotani, 1986; Melki et al. 1989; Walker et al. 1991; Mandelkow and Mandelkow, 1992; Zheng et al. 1995). MTs comprise much of the mitotic spindle apparatus and its proper assembly is essential, as it likely provides the substantial mechanical force for mitotic chromosome segregation (Westermann et al. 2006). Assembly failure of the mitotic spindle generally results in mitotic arrest, apoptosis, and eventually cell death. As such, MTs have become the target for a large number of anti-mitotic agents including antitumor drugs such as the taxanes, epothilones, colchicine and *Vinca* alkaloids, including vinblastine and vincristine (Jordan and Wilson, 2004).

The method of action of these drugs is to either promote or inhibit MT polymerization by binding at specific sites on the surface of α/β-tubulin heterodimers (Figure 1). Paclitaxel, which continues to be one of the most successful cancer therapeutic agents, has a unique mechanism of action as it binds to and results in the stabilization of MTs within all cells (Wani et al. 1971; Schiff and Horwitz, 1980). Derivatives of paclitaxel, such as docetaxel, have been synthesized to address the limited solubility of paclitaxel and show increased binding to β-tubulin (Ringel and Horwitz, 1991). Two additional families of similar tubulin-binding drugs are the epothilones and dolastatins, which share a similar mode of binding. Interest in the epothilones over existing MT stabilizing agents such as paclitaxel is primarily due to their ability to retain cytotoxicity in multidrug resistant (MDR) cells (Bollag et al. 1995). Compounds, such as discodermolide and sarcodictyin have been shown to inhibit the proliferation of human cells through a mechanism similar do that of the taxanes. These compounds seem to selectively stabilize MTs during cell division, and outcompete paclitaxel for tubulin binding (Klein et al. 2005). In the absence of paclitaxel, it has been predicted that the energy stored in longitudinal contacts is greater than that stored in lateral contacts (VanBuren et al. 2002). Recent results using hydrogen/deuterium exchange coupled with mass liquid chromatography have demonstrated that increased rigidity in paclitaxel stabilized MTs was distinct from stabilization as a result of GTP-induced polymerization (Xiao et al. 2006). The authors suggest that paclitaxel increases the overall energy associated with polymerization while maintaining the same differential between lateral and longitudinal contacts in the microtubule lattice. Through structural studies both the taxanes and epothilones were shown to bind a unique site within the β subunit of the α/β-tubulin heterodimer (Lowe et al. 2001; Nettles et al. 2004). Unfortunately, neither of the structures has been able to reveal the precise mechanism of MT stabilization by these drugs.

The *Vinca* alkaloids include vincristine vinblastine and vinorelbine and are used most commonly in combination chemotherapy regimes. Vinblastine was shown to bind at the inter-tubulin dimer interface, ultimately resulting in the net reduction of polymerized tubulin concentration (Gigant et al. 2005). At high concentrations the *Vinca* alkaloid, vinblastine, binds to MTs and results in their depolymerization. However, at low concentrations, vinblastine is thought to bind to MT tips and suppresses their dynamic instability, leading to stabilization (Toso et al. 1993). We feel that this may be a result of vinblastine’s ability to maintain the tubulin dimer at the end of the growing MT in a slightly bent conformation (Wang and Nogales, 2005). If the tip of a protofilament was maintained with a slightly bent conformation, due to the positioning of vinblastine between the dimers, then at low enough concentrations, a small amount of drug bound to the end of the MT would mimic the presence of a GTP cap, thereby resulting in MT stabilization.

The third observed drug site within β-tubulin was shown to bind colchicine, a water-soluble alkaloid that, like paclitaxel, binds to α/β tubulin dimers and blocks cell division thereby inhibiting mitosis (Ravelli et al. 2004). Unlike paclitaxel or vinblastine at low concentration, the binding of colchicine does not result in MT stabilization, but instead results in their destabilization since colchicine binds only to free tubulin and not to tubulin polymerized into MTs.

Following exposure to all of these drugs, cells experience mitotic arrest, which eventually leads to apoptosis. This makes these drugs extremely effective chemotherapeutic agents’ for targeting all rapidly dividing cancerous cells. Nevertheless, even the most successful chemotherapy drugs have undesirable side effects that limit their utility. The anti-tubulin chemotherapeutic agents’ main flaw is that they bind tubulin indiscriminately, leading to the widespread destruction of both cancerous and healthy cells, resulting in unwanted side-effects such as neuropathy (Rowinsky et al. 1989). The ultimate goal of chemotherapy research is to develop a drug or treatment regimen that will target only cancer cells and will target them selectively. In the case of paclitaxel, this would require determining the differences between tubulin as observed in cancerous cells and non-cancerous cells. Fortunately there are slight differences within several β-tubulin isotypes expressed in a range of cell types that may afford a foundation for the development of anti-tubulin drug derivatives with increased specificity for particular cancer cells. Unfortunately, as the precise binding modes of these compounds within β-tubulin is not yet able to fully explain how these drugs affect MT stabilization, our ability to create a precise molecular model of their effects is limited.

### Tubulin isotypes

Many eukaryotic organisms carry multiple genomic copies of functional α or β tubulin, commonly referred to as isoforms (or isotypes if they are confined to a single organism). At the cellular level, the role of tubulin is extremely complex and seems to be related to subtle structural variations observed between the α and β isoforms (Richards et al. 2000). For instance, MT dynamics appears to change significantly as a function of β tubulin isotype expression (Panda et al. 1994). This result implies that if MT assembly/disassembly equilibrium is disrupted through some external stimulus, cells could respond by producing an appropriate isotype mix to restore normal balance. The existence and varied distribution of tubulin isoforms provides a link to their function in the polymerization and stability of MTs. While the presence of numerous tubulin isoforms suggests that they may play specific roles in MT function, there are no precise predictive models to describe differences between them.

**Figure 2.**
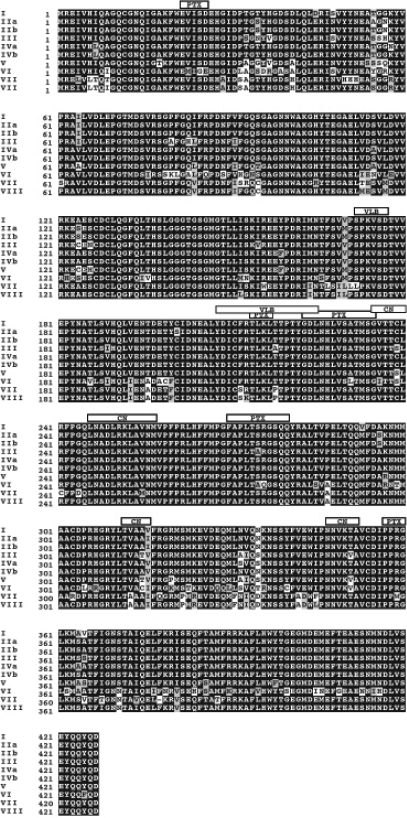
Sequence alignment of β tubulin isoforms. Each of the ten Human β-tubulin isoforms that was identified in our screen of the Uniprot and NCBI Entrez databases was aligned using the ClustalW software package (Thompson et al. 1994). Prior to performing the alignment, the highly variable carboxy terminal residues were removed from each sequence. This was done as the structural file, 1JFF, does not contain any of these residues. At each position within the alignment, black boxes indicate identical residues, grey boxes indicate residues that are conserved, while white boxes indicate residues that are divergent. The 6 Å cutoff binding sites for paclitaxel, colchicine and vinblastine are indicated by boxes labeled PTX, CN and VLB, respectively.

In humans, several α and β tubulin isotypes have been identified and characterized (Lu and Luduena, 1994; Luduena, 1998; Roach et al. 1998). While α tubulin must play an obvious role in the determination of MT function, we have chosen to focus only on β tubulin for this discussion, as most of the available data in the literature deals with this protein as a target for drug action and protein-protein interactions. Through a search of available protein sequence databases, we have previously identified a total of ten unique β tubulin isotypes, all of which have related amino acid sequences and are generally well conserved (Huzil et al. 2006). Despite having similar sequences, specific regions of higher sequence variability have been identified (Figure 2). The highest degree of sequence variability between the α and β-tubulin isotypes occurs in the extreme carboxy terminal region (residue 430 and greater). This C-terminal region has been used to identify distinct β-tubulin isotypes, based on their reactivity with monoclonal antibodies (Banerjee et al. 1990; Panda et al. 1994).

Each β-tubulin isotype seemingly has a unique pattern of expression ranging from highly specific for classes III, IVa and VI, to constitutive expression for classes I and IV (Cowan and Dudley, 1983; Jensen-Smith et al. 2003). Class I β-tubulin is the most commonly expressed isotype in humans and as such is also the most common isotype found in cancer cells (Sullivan and Cleveland, 1986). Both classes II and III β-tubulin have been observed at increased levels in human tumors (Scott et al. 1990; Ranganathan et al. 1996; Kavallaris et al. 1997; Prasannan et al. 2000; Dozier et al. 2003; Ferguson et al. 2005; Mozzetti et al. 2005).

Recently, the role of β-tubulin isotypes in resistance to anti-mitotic drugs has become a topic of great interest, as the design of novel drugs based on isotype specific differences would result in better treatment protocols (Burkhart et al. 2001). However, in addition to functional tubulin isotypes, the tubulin gene family also contains several pseudo-genes, which are non-functional sequences within the genome having close similarities to functional genes. There are several human β-tubulin pseudo-genes, all of which show substantial homology to the Class I β-tubulin gene, a situation which has resulted in the erroneous identification of several tubulin mutations that correlate with paclitaxel resistance (reviewed by Berrieman et al. 2004). This has added controversy to an otherwise attractive hypothesis that mutations within the β-tubulin genes can lead to drug resistance.

### Drug resistance mechanisms involving tubulin

As with all chemotherapeutic treatments, the development of drug resistance to anti-tubulin agents is a major clinical problem with no simple solution. Drug resistance can originate through several mechanisms involving: (a) changes in cellular drug uptake, (b) drug metabolism, (c) structural changes in the drug target, (e) drug efflux, or (f) changes in cellular components that interact with the target. Cell culture studies have previously demonstrated that the most frequent mechanism for resistance to drugs such as the colcemides and *Vinca* alkaloids is P-glycoprotein mediated multidrug resistance (Cabral and Barlow, 1989; Dumontet et al. 1996). However, in direct contrast to this observation, cells which show resistance to taxanes tend to exhibit alterations in tubulin expression patterns with low P-glycoprotein activity. In addition to the altered expression of β-tubulin isotypes, point mutations in tubulin leading to alterations and expression related post-translational modifications of tubulin regulatory proteins, such as stathmin, microtubule associated protein (MAP), tau and MAP4 have also been implicated in changes to MT dynamics and the development of drug resistance (Liu et al. 2001).

In addition to isotype expression and acquired mutations, tubulin is also thought to undergo an auto-regulatory mechanism where treatment with MT destabilizing drugs, such as colchicine, will ultimately result in the coordinated degradation of tubulin mRNA and a corresponding reduction of cellular free tubulin dimers. The synthesis of α and β-tubulin monomers appears to be regulated by a mechanism in which α-tubulin translation is inhibited by free α-tubulin but not by α-tubulin that is complexed with the β-subunit (Gonzalez-Garay and Cabral, 1996). In this way, β-tubulin is synthesized only when there are available α-subunits to which it can bind. The treatment of cells with MT stabilizing drugs, such as paclitaxel results in the overall stabilization of tubulin mRNA and an increase in tubulin protein due to a net reduction in concentration directly following drug treatment. This results in the overall equilibrium of free tubulin within the cell and maintains a steady supply of tubulin for MT synthesis (Boggs and Cabral, 1987; Theodorakis and Cleveland, 1992; Barlow et al. 2002). This further implies that any mutation or expression of a tubulin isotype that affects the overall stability of MTs will also have an effect on the total amount of free tubulin dimers that are present within the cell.

Finally, tubulin can also be controlled post-translationally through degradation. Although tubulin heterodimers are very stable (Spiegelman et al. 1977), excess free subunits (Gonzalez-Garay and Cabral, 1995) or defective tubulin proteins have a much shorter half-life (Kemphues et al. 1982; Boggs and Cabral, 1987). The tubulin molecule is also subject to several post-translational modifications which are thought to also affect their rate of assembly into MTs. Some of these modifications, such as phosphorylation and acetylation are common to many proteins. Others, such as polyglutamylation, are very rare, while still others, such as tyrosinolation/detyrosinolation and polyglycylation, have so far been reported only in tubulin (Luduena, 1998; MacRae, 1997; Westermann and Weber, 2003; Soucek et al. 2006).

### Drug resistance due to β-tubulin isotype expression levels

Several studies have pointed to the over-expression of tubulin isotypes as a possible mechanism resulting in drug resistance ( Banerjee et al. 1990; Minotti et al. 1991; Banerjee and Luduena, 1992; Haber et al. 1995; Kavallaris et al. 1997; Derry et al. 1997; Banerjee and Kasmala, 1998; Ranganathan et al. 1998; Verdier-Pinard et al. 2003). While other studies suggest that expression of certain tubulin isotypes does not lead to paclitaxel resistance (Blade et al. 1999; Nicoletti et al. 2001; Sale et al. 2002). However, as more data is acquired, it is becoming clear that isotype expression does lead to drug resistance at some level. In particular, Class III β-tubulin has been implicated in paclitaxel resistance through a mechanism that results in decreased MT stability, thus counteracting the effect of paclitaxel (Sangrajrang et al. 1998; Katsetos et al. 2003; Hari et al. 2003a; Shalli et al. 2005). More specifically it appears that the expression of specific tubulin isotypes, such as βIII, may not affect MT dynamic instability in the absence of drugs such as paclitaxel, but may act to suppress the drug’s ability to affect MT dynamics directly (Kamath et al. 2005). Finally, support for the role of tubulin isotype expression and their effect on drug resistance was observed when antisense oligonuclotides specific for βIII result in the resensitization of resistant cells to paclitaxel (Kavallaris et al. 1999). In addition to the several observations of tubulin isotype expression patterns in tumor cells, the expression of tubulin isotypes has also been implicated in the differentiation state of cells (Carles et al. 1999).

**Figure 3.**
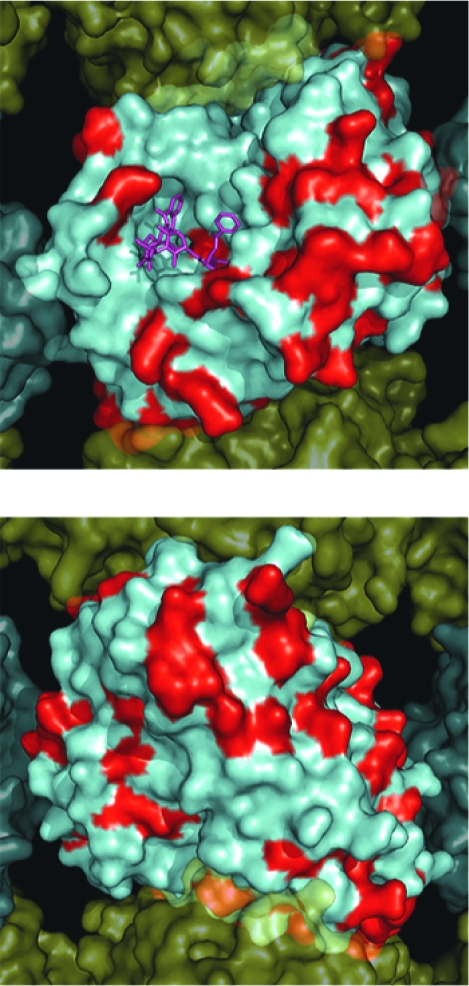
Surface map of nonconserved residues in all Human β-tubulin isoforms. A solvent accessible surface was drawn onto the α/β-tubulin heterodimer obtained by Nogales et al. 2001 (PDB identifier 1JFF). This figure shows an α/β-tubulin heterodimer within a MT containing 13 protofilaments which was reconstructed as described by Li et al. (2002). The α-tubulin surface is colored yellow and the β-tubulin surface colored cyan with nonconserved positions colored red. **Panel A.** Illustrates the MT from the interior surface and shows the binding site for paclitaxel (purple). **Panel B.** Illustrates a 180° rotation about the y-axis to show the exterior surface of the microtubule **A**. β-Tubuiln Isotypes MT Interior **B**. β-Tubuiln Isotypes MT Exterior

We have previously described the construction of a complete set of homology models for the human β-tubulin isotypes and described several differences that we observed between them (Huzil et al. 2006). Sequence alignment of all the β-tubulin isotypes demonstrates that many of the differences between them occur outside the main drug binding sites (Figure 2). When displayed on the surface of the 3D tubulin structure, it becomes apparent that most of the differences found between the various isotypes are restricted to the lateral and longitudinal surfaces that are involved in protein-protein interactions with the neighboring tubulin dimers in the MT (Figure 3). Differences at these locations may provide the cell with the ability to alter expression patterns for tubulin isotypes and in this way affect the overall kinetics of MT assembly and disassembly. This could be in response to such external stimuli as temperature changes or drug exposure. Interestingly, several differences can be observed on the exterior face of the β-tubulin when it is assembled into the MT, while there are significantly fewer observed differences on the inner surface. This may be a result of selective differences between isotypes that are meant to accommodate Microtubule Associated Protein (MAP) binding, thereby leading to altered MT kinetics (Kurz and Williams, 1995).

The underlying molecular mechanisms of tubulin isotype expression, in particular Class III β-tubulin over-expression, in drug resistance are not well understood. There are a number of possibilities, including tubulin’s normal vibrational modes, perhaps resulting in altered protein dynamics. More likely, it is a critical change within surfaces that interact during MT assembly. Therefore, the expression of specific tubulin isotypes could result in the alteration of MT stability. A cellular requirement for less stable MTs may then result in the over-expression of isotypes such as βIII, which has been demonstrated to be produce more dynamic MTs than other isotypes (Derry et al. 1997).

### Tubulin mutations and drug resistance

The first studies that identified resistance to antimitotic drugs through a mechanism of β tubulin mutation were originally performed in the early 1980’s (Ling et al. 1979; Cabral et al. 1980; Keates et al. 1981; Cabral et al. 1982; Cabral and Barlow, 1989). However, the work of Monzo et al. (1999) in observing mutations of β-tubulin in human lung cancer cell lines has been the source of much controversy since its publication. It has since been suggested that the presence of tubulin pseudo-genes and sequencing errors result in erroneous interpretation and therefore makes determining true mutations in tubulin difficult. Many sequencing errors within the β-tubulin gene tend to be a result of primer selection where intronic primers are used or pseudo-genes are inadvertently sequenced (de Hasegawa et al. 2002; Tsurutani et al. 2002; de Castro et al. 2003; Achiwa et al. 2003; Maeno et al. 2003; Berrieman et al. 2004; Mesquita et al. 2005).

It has been suggested that differences in drug binding affinities do not play a significant role for the colcimides or vinblastine with regards to the acquisition of tubulin mutations in certain cell lines (Hari et al. 2003b). However, there are several studies, discussed below, that point to a role for mutations within the taxane binding site as a mechanism for resistance to paclitaxel. We feel that mutations observed within the β-tubulin genes may not result in changes to drug binding affinity, but will more likely play a role at distant locations within the protein. The rarity of true somatic mutations in tubulin in clinical samples also suggests that other mechanisms, such as drug efflux or changes in the expression levels of the different β-tubulin isotypes, are a more important contributing factor for anti-tubulin drug resistance (de Castro et al. 2003; Maeno et al. 2003; Hasegawa et al. 2002; Ferguson et al. 2005). However, the positive data obtained with cell lines suggests that further investigation is needed to ascertain what role, if any, mutations in the β-tubulin gene have in predicting clinical response to anti-tubulin agents. We cannot discount the role of intronic mutations and their observed role for drug resistance and how this may ultimately affect gene expression of a particular tubulin isotype as de Castro et al. (2003) observed that as alterations in β-tubulin were amplified with exonic primers less clinical response to paclitaxel was observed. Additionally, observations made with regards to acquired mutations are often complicated by the concurrent overexpression of different tubulin isotypes, making the interpretation of results difficult.

## Analysis of Specific β-Tubulin Mutations

**Figure 4.**
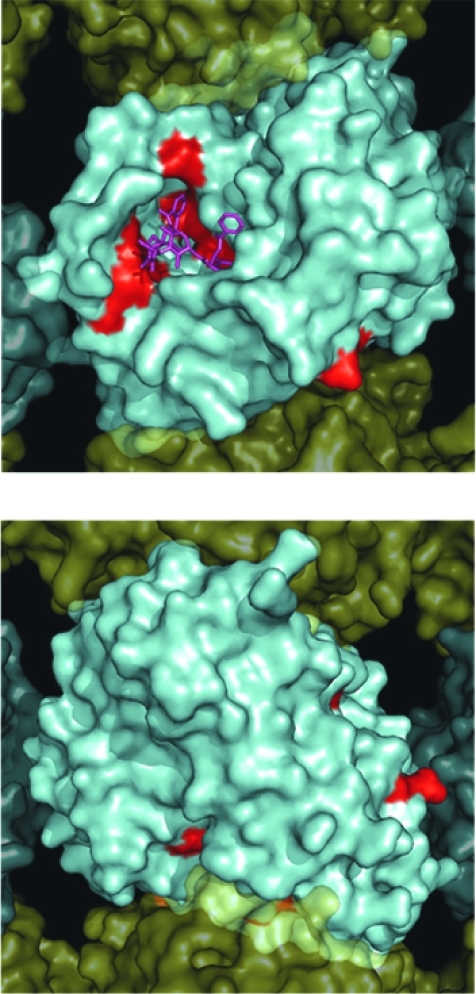
Surface map of mutation sites in mammalian β-tubulin. As in Figure 3, a solvent accessible surface was drawn onto the α/β-tubulin heterodimer and assembled into a 13 protofilament MT. Here the β-tubulin surface is colored red to illustrate the surface positions of mutations observed in Table 1. **Panel A.** Illustrates the MT from the interior surface and shows the binding site for paclitaxel (purple). **Panel B.** Illustrates a 180° rotation about the y-axis to show the exterior surface of the microtubule. **A**. β-Tubuiln Mutations MT Exterior **B**. β-Tubuiln Mutations MT Exterior

**Figure 5.**
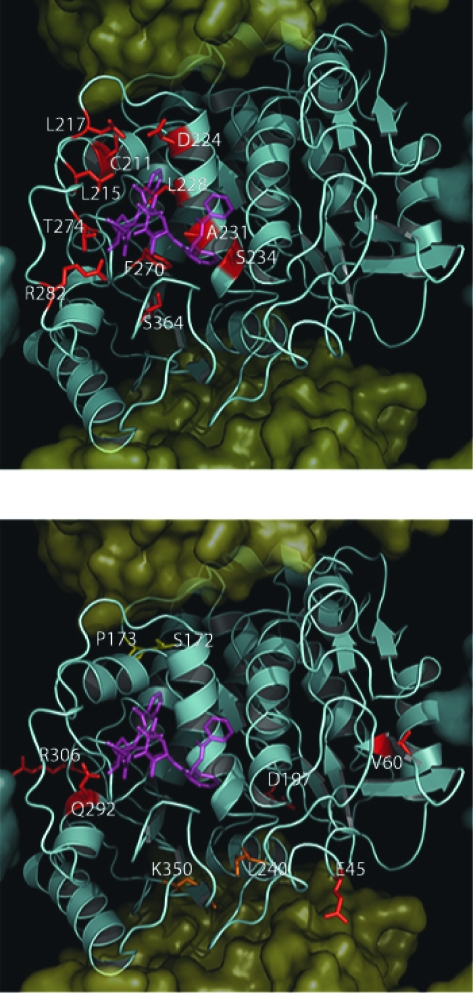
Location of mutations in mammalian β-tubulin in the taxane binding site. As in Figure 3, with the exception that the β-Tubulin surface has been removed to expose the underlying tertiary structure which is shown as a cyan cartoon. **Panel A.** Shows mutations found within the taxane Binding Site (C211, L215, L217, D224, L228, A231, S234, T274, F270, R282, S364) colored red in relation to paclitaxel (purple). **Panel B.** Shows residues found outside the taxane binding site (E45, V60, Q292, R306) colored red. Residues found within the *Vinca* binding site (S172 and P173) are colored yellow and residues found within the colchicine binding site (L240 and K350) are colored orange. **A**. β-Tubuiln Mutations Within PTX Site **B**. β-Tubuiln Mutations Not In PTX Site

**Figure 6.**
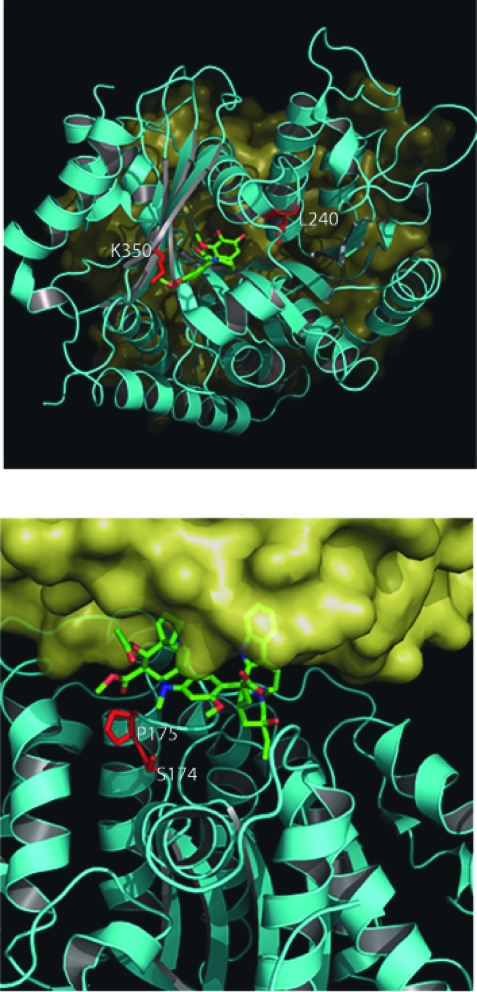
Location of mutations in mammalian β-tubulin in the colchicine and *Vinca* binding sites. As in figure 5, the surface has been removed from β-tubulin. **Panel A.** Shows a view of the colchicine binding site from the longitudinal surface between an α/β-tubulin heterodimer. The α-tubulin has been removed to facilitate viewing of the bound colchicine within the β-tubulin a second α-tubulin can be seen behind β-tubulin in the background. Colchicine is colored green and is shown as sticks. The two mutated residues (L240 and K350) are shown as red sticks. **Panel B.** Shows a view of the Vinca binding site from the lateral surface between two protofilaments. The vinca alkaloid vinblastine is shown as green sticks and the two mutated residues (S174 and P175) are shown as red sticks. **A**. Colchicine Binding Site Longitudinal Surface **B**. Vinblastine Binding Site Lateral Surface

To determine which residues within each of previously described drug binding sites within β-tubulin are essential for activity, we examined the location of reported mutations that confer resistance to tubulin-binding drugs in all reported mammalian cells (Table 1). There seemingly is little correlation between the overall structure of the paclitaxel binding site and the location of mutations in β-tubulin that occur as a result of resistance following exposure to paclitaxel (Figure 4). We have observed that within the set of 21 unique positions within the tubulin sequence, only eleven mutations were found to be contained within the taxane binding site (Figure 5a). Only eight of these substitutions were identified as becoming either paclitaxel or epothilone resistant. Interestingly three of the mutations within the taxane binding site resulted in MT stabilization and as a result became resistant to either colchicine or vinblastine. Of the remaining ten sites within β-tubulin, five were shown to become paclitaxel resistant, yet did not occur in the vicinity of the taxane binding site (Figure 5b). Of two sites found near the colchicine binding site, only K350N was identified as being colchicine resistant (Figure 6a) (Hari et al. 2003b). Interestingly, mutation L240I, which is also found near the colchicine binding site, confers resistance to vinblastine (Kavallaris et al. 2001). This was a remarkable observation as the *Vinca* binding site is on the opposite face of β-tubulin from the colchicine binding site (Figure 1). There are also two mutations situated near the *Vinca* binding site. The first at S172 conferred vinblastine resistance (Poruchynsky et al. 2004) and the second, at P173 curiously conferred epothilone resistance with an opposing MT destabilizing phenotype (Figure 6b) (He et al. 2001).

### Mutations found within the taxane binding site

When examining mutations acquired following exposure to taxanes or epothilones, several are found directly within the taxane binding site and could therefore affect drug binding through VDW (Van der Waals (VDW) interactions, electrostatics or hydrophobic differences. These include C211, L215, L217, D224, L228, A231, S234, F270, T274 and R282 A364 (Figure 5a). We should note that while all of these residues are located within close proximity of the bound paclitaxel molecule from the 1JFF structure, most were reported not to change the affinity of the taxanes or epothilones, but appear to destabilize MTs in the absence of any drug (Gonzalez-Garay et al. 1999; Verrills et al. 2003; Hari et al. 2003b). Of these eleven residues found to be directly in contact with either paclitaxel or epothilone, only F270V, T274I and R282N were reported to have a direct effect on drug binding affinity (Giannakakou et al. 1997; Giannakakou et al. 2000). This observation suggests that there must be a global effect on tubulin dimer interactions that are involved in MT assembly rather than a reduced paclitaxel binding effect as the mechanism for drug resistance.

**C211/D224/S234.** Substitutions at C211, D224 and S234 all were all shown to result in resistance to both colchicine and vinblastine, producing an increase in MT stability and the amount of polymerized MT observed within cells (Hari et al. 2003b). All of these substitutions are located within a structure known as the M loop, which has been shown to be critical for the function of paclitaxel and MT dynamics, affecting lateral interactions between tubulin subunits in the MT (Figure 5a) (Keskin et al. 2002). The significance of this region in MT assembly is illustrated, not only by the fact that both the paclitaxel and colchicine binding sites are nearby, but also because many mutations conferring resistance to drugs that either promote or inhibit MT assembly are also located in this same area (see following discussion). This is an interesting result, as these residues are clearly located near the taxane binding site and are within 8–10 Å of bound paclitaxel. However, their proximity to both the GTP binding site and the colchicine binding site may also result in alternate phenotypes depending on the substitutions. This is supported by the observation that mutations at these positions in yeast were shown to confer variable drug resistance depending on the amino acid substitution indicating the critical role that this region plays in MT stability and drug resistance (Gupta et al. 2001).

**L215, L217, L228.** Analysis of β-tubulin alleles from nine paclitaxel-resistant Chinese Hamster Ovary (CHO) cell lines revealed a cluster of mutations affecting L215, L217, and L228 (Gonzalez-Garay et al. 1999). At position 215, histidine, arginine, or phenylalanine substitutions were observed, while at position L217, an arginine and at L228 either histidine or phenylalanine substitutions were observed. All of these mutations resulted in a decrease in MT stability, as demonstrated by an overall reduction in the level of polymerized tubulin within the cell and by increased resistance to paclitaxel. Substitutions at positions L215 and L217 are in a loop that connects helices H6 and H7, while L228 is located within H7 itself (Figure 5a). These substitutions may produce resistance as a result of their location in or near H7 since it has been hypothesized that H7 controls the conformation of the entire molecule (Amos and Lowe, 1999). An interesting observation was made by Wang et al. (2006) when the substitution L215V did not produce any observable phenotype while mutation to any other residue produced cells resistant to paclitaxel as a result of global MT destabilization. Perhaps the specific spatial arrangement of the two methyl groups found on leucine and valine results in no observable effect for this mutation. However, this observation requires additional experimentation before a concrete mechanism can be established for this apparent selectivity.

**T274/R282.** In addition to describing a common pharmacophore for Epo A/Epo B binding which may explain mutational effects at L215, L217 and L228, Giannakakou et al. (2000) also identified mutations at positions T274 and R282 as conferring either epothilone or paclitaxel resistance. Both of these mutations resulted in a similar reduction in the overall amount of polymerized tubulin within the cell. While these mutations did seem to have an effect on the ability of tubulin to bind either epothilone or paclitaxel, they also conferred a greater degree of MT destabilization, which is most likely the major factor leading to drug resistance. Both of these residues are found within the M loop and their interactions with the taxane site are direct and most likely significant (Figure 5a). As the amino terminus of the M loop comprises part of the taxane ring-binding region, a mutation of R282 could directly affect the binding of both the taxanes and epothilones. However, as previously discussed, the involvement of the M loop within longitudinal MT interactions is extremely important for proper assembly and stability. The importance of this region is further supported by the observation that both T274 and R282 are evolutionarily conserved in all known human β-tubulin isotypes (Figure 2).

The C4–C5 oxetane of the bound taxane is located near the polar backbone atoms of residues 273, 275, 276 and the hydroxyl side chain of T274. Therefore, substitutions of this residue may lead to a change in the hydrophillicity of this region making taxane binding less favorable. Additionally, Giannakakou et al. (2000) also hypothesized that cross-resistance to both epothilone and paclitaxel may be a result of the epothilone C7-OH hydrogen bond with the vicinity of T274. Therefore, T274I results in the loss of this hydrogen bond. In contrast, paclitaxel has hydrogen bond donors or acceptors at their C10, C9, and C7 positions and can presumably form alternate hydrogen bonding patterns.

**F270V, A364T.** Cell cultures resistant to paclitaxel but not epothilone and not dependent on paclitaxel for growth, suggested that the mutation F270V is specific only for paclitaxel binding and does not lead to an overall reduction in MT assembly kinetics (Giannakakou et al. 2000). However, collateral sensitivity to vinblastine suggests a global destabilization of the MT structure also. This mutation was independently selected along with another mutation, A364T, which also demonstrated the same paclitaxel, but not epothilone-resistant phenotype. Both of these substitutions occur within the taxane binding site and comprise the majority of the surface that makes up the floor of this binding site (Figure 5a). Several possible interactions with the taxane C13 side chain are possible. Substitution of A364T would result in a polar residue in this normally hydrophobic surface. While F270 does not seem to participate in any significant ring interactions, this effect cannot be ruled out as it is in close proximity to both C13 phenyl rings. The resistance to paclitaxel but not epothilone for both of these substitutions suggests that there is a difference in the binding pocket that does not affect epothilone binding. This effect may be due to the epothilone molecule sitting higher within the binding pocket at this region and/or due to the lack of potential aromatic interactions with F270.

**A231.** Through screening a series of desoxyepothilone B resistant leukemia cell lines, the mutation A231T was seen to be producing less stable MTs (Verrills et al. 2003). This substitution was shown to confer resistance to the epothilone analog desoxyepothilone B, but did not seem to alter drug binding. The authors also observed that cells carrying this mutation were hypersensitive to microtubule destabilizing agents and expressed increased levels of class III β-tubulin. These cells also characteristically had a greatly reduced tubulin polymer mass which is indicative of reduced MT stability. The A231T substitution is located on H7 within the highly hydrophobic region of the taxane binding site (Figure 5a). Substitution of a hydrophobic alanine to a polar threonine residue should produce a significant effect on drug binding. However, as there was no observable effect on paclitaxel binding, the authors suggested that the change was more likely to result in the alteration of the conformation of the H7, thereby affecting MT stability through a reduction in the α helical propensity associated with threonine over alanine.

**Q292.** Cells carrying a Q292E, H mutation were shown to become resistant to epothilone and paclitaxel probably as a result of reduced drug binding (He et al. 2001; Verrills et al. 2003; Yang et al. 2005). This mutation was originally identified as a suppressor of the mutation D45Y, originally identified by Wang et al. (2004). In the presence of the mutation A231T, which was not shown to affect paclitaxel binding directly, mutations at position 292 were shown to reduce paclitaxel binding (Verills et al. 2003). As both of these mutations resulted in reduced MT levels, the mutation of residue 292 may have a direct influence on paclitaxel binding. Interrestingly, as can be seen in Figure 5a Q292 is not in direct contact with the bound paclitaxel molecule, but is located on H9 near the M loop, which is implicated in the critical lateral contacts within the MT. This residue is also approximately 7.5 Å from the four-membered oxetane ring within paclitaxel or the ketone oxygen within epothilone and may therefore affect the binding pocket indirectly. Additionally, both glutamine and glutamic acids prefer solvent exposed conformations, they are frequently observed at protein interfaces. The resulting change from glutamine to glutamic acid introduces a negative charge which may decrease protofilament-protofilament interactions at this surface. This hypothesis is supported by the observation that cells carrying this mutation are sensitive to treatment with MT destabilizing drugs such as vinblastine. When the Q292E was expressed in combination with V60P cells became dependent on epothilone for growth (Yang et al. 2005). Both mutations at Q292 and V60 would be expected to impact lateral contacts between protofilaments. Therefore, cells carrying these mutations would require MT stabilizing drugs, such as paclitaxel or epothilone, to compensate for the increased instability of their MTs. Because of its close proximity to the taxane binding site, this mutation may have an effect on drug binding, although this is not likely.

### Mutations within the colchcicine binding site

**L240.** The L240I mutation was identified in tumor cell lines that became resistant to the MT destabilizing drug vincristine as a result of increased MT stability (Kavallaris et al. 2001). This mutation lies in a region of β-tubulin that is in close proximity to the α/β-tubulin interface on the loop that links H7 and H8 (residues 238–250) (Figure 6a). A mutation at this position would affect longitudinal interactions between α and β monomers thereby stabilizing MTs conferring its observed *vinca* resistance phenotype. The authors proposed that a leucine-to-isoleucine substitution at this position is close to alanine 248 and may result in a conformational change within H7. As discussed previously, H7 lies at the base of the taxane binding site and has been implicated in the global stability of the tubulin dimer (Amos and Lowe, 1999). This would support the observation that cells carrying this mutation also had a concurrent increase in polymerized tubulin. Due to its distance from the *Vinca* binding site, this mutation was hypothesized not to have an effect on drug binding, but is thought to lead only to increased MT stability.

**D350.** See the D197/K350 discussion below.

### Mutations within the *Vinca* binding site

**S172.** A single mutation at position S172 resulted in an overall increase in MT stability and a resulting resistance to the hemiasterlin HTI-286 (Poruchynsky et al. 2004). This mutation also produced cross-resistance to vinblastine binding site agents, as the hemiasterlins have been proposed to share a binding region on tubulin with the vinca alkaloids (Bai et al. 1999). This mutation was shown to affect polymerization through the stabilization of MT thereby producing resistance to destabilizing and sensitivity to polymerizing drugs. The main reasoning for this may be due to the fact that S172 has recently been shown to be a substrate for phosphorylation by cyclin-dependent kinase Cdk1 at the transition from interphase to mitosis (Fourest-Lieuvin et al. 2006). The phosphorylation of this Serine residue was shown to decrease MT stability and its loss would effectively stabilize MTs, thereby making them more resistant to destabilizing drugs. Therefore, resistance may be due an increase in stability or may also be a result of reduced drug binding due to its proximity to the *Vinca* binding site.

Interestingly, mutation of P173A has the opposite effect leading to MT destabilization and resistance to epothilone and sensitivity to colchicine (He et al. 2001). This difference may be due to a catastrophic conformational change in the T5 loop as a result of the loss of P173. Neither of these mutations was shown to have any effect on drug binding.

**P173.** Mutations at P172 were shown not to affect the binding of Epothilone and have a destabilizing effect on MT structure in HeLa cells (He et al. 2001). P173 is located on the T5 loop which, as just described, is involved in forming the ribose binding component of the nucleotide binding pocket (Figure 6b). It is highly probable that P173 acts to stabilize this loop and is, therefore, essential for proper nucleotide orientation within the nucleotide binding pocket. This would then have a direct effect on nucleotide binding and subsequent hydrolysis, resulting in altered MT dynamics. Interestingly, while this mutation occurs next to S172, it has the completely opposite effect, resulting in the destabilization of MTs and a resistance to MT stabilizing drugs.

### Mutations outside the drug binding sites

**D45.** The mutant D45Y was shown to confer growth dependence on colcemid as a result of MT stabilization (Wang et al. 2004). This mutation caused an increase in the levels of polymerized tubuiln found within the cell, supporting the stabilization of MTs. This increase in stability could, therefore, explain the resistance to drugs such as colchicine and vinblastine which destabilize MTs and the increased sensitivity to those drugs like paclitaxel and epothilone that cause MT stabilization. This is most likely a result of D45 location within a loop connecting helix 1 (H1) with β-sheet 2 (S2), which was predicted to be a critical contact between tubulin dimers within protofilaments (Figure 5b) (Li et al. 2002). This position is not close to the site where colcemid binds to tubulin and it is, therefore, unlikely to act by changing the affinity of tubulin for the drug. Wang et al. (2004) also identified several revertants of D45Y MT stabilization effects. V60 is also located in the H1-S2 loop and its replacement with alanine results in the reduction of MT assembly rates, thereby conferring resistance to paclitaxel. These authors observed that when D45Y and V60A substitutions are co-expressed, their individual effects on MT assembly result in a normal phenotype. Because both of these substitutions are found at a critical location for protofilament assembly, it is not surprising that they display such a marked effect on MT stability. An additional suppressor of the D45Y phenotype, Q292 was also identified by Wang et al. (2004) to confer resistance to paclitaxel. This mutation resulted in the severe destabilization of MTs and is located in H9, which is positioned near the M-loop which is involved in MT lateral interface interactions and the taxane binding site (Figure 5b). This position was also shown to become mutated within human tumor cell lines that became resistance to desoxyepothilone B and epothilone B (Verrills et al. 2003; Yang et al. 2005).

**V60.** As discussed above, mutations at V60 were observed under two separate studies which-both resulted in decreased MT stability and reduced assembly rates and a requirement for paclitaxel or epothilone presence for growth (Wang et al. 2004; Yang et al. 2005). This mutation was originally identified as a suppressor of D45Y. Residue V60 is located at the end of the H1-S2 loop that has been implicated as a principal partner of the M loop for contacts between protofilaments (Figure 5b). A mutation at residue 60 could potentially inhibit the lateral contacts between protofilaments, resulting in the destabilization of MTs and is not predicted to confer altered drug binding.

**D197N/K350N.** 2-Methoxyestradiol is a potent anticancer agent that is thought to result in the destabilization of MT dynamics through its binding at the colchicine binding site (D’Amato et al. 1994). Cells expressing both the D197N and K350N mutations simultaneously became resistant to 2-Methoxyestradiol, colchicine and vinca alkaloids (Gokmen-Polar et al. 2005). This observation was suggestive of a global stabilization of the MT structure with no effect on drug binding, due to the different binding sites for vinblastine and colchicine. Additionally, while these cells were resistant to MT destabilizing drugs, they did not appear to be sensitive to MT stabilizing drugs such as Taxol.

Several interactions for K350 and D197 illustrate a possible structural basis of the resistance to 2-methoxyestradiol when either is mutated to asparagine. First, K350 is not only located near the colchicine binding site, but it also makes critical longitudinal contacts with α tubulin as it is buried at the intra-dimer interface (Figure 6a). Large differences in IC50 values for both colchicine and vinblastine binding also suggested that drug binding was not affected as these two compounds bind at different sites within β-tubulin (Gigant et al. 2005). K350 may play a minor role in stabilizing the α-phosphate moiety of GTP bound tubulin, as it is only 6–7 Angstroms away. Because it is in close proximity to the colchicine binding site, K350’s participation in direct interactions with colchicine cannot be ruled out entirely.

Unlike K350, which forms bonds with portions of α-tubulin, all of the local interactions for D197 reside within β tubulin itself. D197, which resides at the end of S6, participates in a series of hydrogen bonds that are identical in both the straight and curved conformations of tubulin (Figure 5b) (Wang and Nogales, 2005). None of these hydrogen bonds would be significantly disrupted by the mutation of D197N. However, D197 does participate in a salt bridge with R156 within H4. Removal of this salt bridge would most likely lead to a loss of anchoring of H4 and alter the propagation of conformational changes within β-tubulin, thereby influencing MT stability.

**R306.** Substitutions at position R306 were identified in cells from paclitaxel resistant tumors (Hasegawa et al. 2002). While this substitution did result in resistance to paclitaxel, there was no determination if this was due to the destabilization of the MT structure, or a direct impact on drug binding. However, this substitution lies within the longitudinal interface between protofilaments and interacts with residue D118 within the adjacent β-tubulin monomer (Figure 5b). This interaction could constitute an additional salt bridge that acts to stabilize the longitudinal interface between protofilaments. Substitution to threonine would result in the destruction of this interaction and could very possibly lead to an overall decrease in MT stability, thereby producing a mechanism of resistance to paclitaxel.

**Y442.** Identified as a heterozygous mutation, most likely due to a lethal phenotype in the homozygous case. Substitution of Y442C resulted in cells that were dependent on either paclitaxel or epothilone for growth (He et al. 2001). This observation is, once again, suggestive of a global change in MT stability and not simply a decrease in overall drug binding. This substitution occurs within the end of H12 in a region not resolved in the crystal structure of the α/β-tubulin heterodimer (Lowe et al. 2001). It is generally accepted that this region makes up the most variable part of the tubulin isotype sequences and is commonly referred to as the C-terminal tail. This substitution is therefore most likely associated with the alterations of interactions with MAPs or proteins involved in the post translational modification of the C-termini (Luduena, 1998).

## Correlation Between β-Tubulin Isotype Expression and Mutations

**Figure 7.**
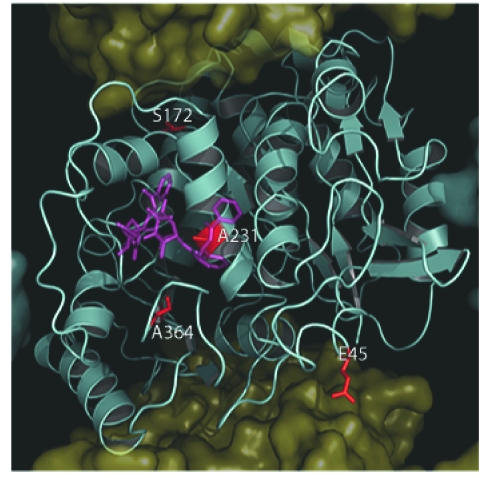
Location of mutations and nonconserved residues in mammalian β-tubulin. As in Figure 3, with the exception that the β-Tubulin surface has been removed to expose the underlying tertiary structure which is shown as a cyan cartoon. Here residues (F45, S172, A231 and A364), which are observed as occurring in both human β-tubulin isotypes and mutations identified in Table 1, are shown as red sticks in relation to paclitaxel (purple). Correlation Between β-Tubuiln Isotypes and Mutations

We have examined the correlation between the positions of sequence differences associated with β-tubulin isotypes and the mutations described here. Of all 21 mutations, only four seem to correlate with residue differences between β-tubulin isotypes (Table 2, Figure 7). The first, at residue 45, is a conservative substitution from aspartic to glutamic acid and would most likely not affect MT dynamics in the class βII–VIII, isotypes that carry it. Next within the human isotypes at position 172, we observed the presence of a normal serine residue in class βI–VI and βVIII isotypes. However, in class βVII this serine has been replaced by a leucine. As described above, this substitution results in the loss of a polar serine. As this residue has been shown to be phosphorylated during cell division, its loss would lead to the resulting stabilization of MTs into which it was incorporated (Fourest-Lieuvin et al. 2006). While the expression of class βVII has yet to be correlated with resistance to MT destabilizing compounds or increased MT stability, this is an avenue of experimentation worthy of future investigation. The mutant A231T was shown to confer paclitaxel or epothilone resistance onto cells that expressed it (Verrills et al. 2003). Cells expressing this mutation were also shown to exhibit greater levels of the class βIII isotype expression. As previously stated, the A231T substitution is located on H7 within a highly hydrophobic region of the taxane binding site. In the case of class βVI isotype containing a leucine at position 231, we expect that this conservative substitution would not lead to a dramatic change in phenotype.

Finally, we feel that one of the most significant observations we have made with regard to the correlation between an isotype sequence and an observed mutation is the presence of an A364S substitution within the class βIII and βV isotypes. A364T was shown to become resistant to paclitaxel but not epothilone (Giannakakou et al. 2000). This substitution occurs within the taxane binding site and makes up a portion of the hydrophobic floor upon which paclitaxel and docetaxel’s C13 side chain sits. The substitution of A364S in either class βIII or βV isotypes would result in a similar situation to that for the A364T mutant, a polar residue in this normally hydrophobic surface. This observation is exciting as the A364T mutation alone was shown to impart selective paclitaxel resistance. This, coupled with the observation that the expression of the class βIII tubulin isotype may be correlated with paclitaxel resistance, points to a possible mechanism and target for this resistance (Mozzetti et al. 2005).

## Conclusions

Even with the vast quantity of data available to us, it is still not entirely clear what effect the expression of a particular β-tubulin isotype, or acquisition of point mutations has on the stability of cellular MTs and their relationship to drug resistance. Two possible models may explain the role that these two processes play in drug resistance. First, it may be possible that certain isotypes or mutations exhibit decreased drug binding to β-tubulin. However, our previous observations suggest that while this may contribute to drug resistance, it most likely plays a minimal role due to the lack of observable differences at the drug binding sites (Huzil et al. 2006).

A second mechanism would produce global conformational differences between β-isotypes or mutants, thereby attenuating MT dynamics, making them more or less stable. We feel that the most interesting observation that has come out of this examination of mutations within β-tubulin is that out of a total of fifteen residues that are found within the binding sites for either taxanes, colchicine or vinca alkaloids, only five were definitively identified as having a direct impact on drug binding. Although the mutations we have identified are close to the region implicated in the binding of paclitaxel, a number of observations indicate that altered paclitaxel binding is not responsible for drug resistance in these mutants. For example, β-tubulin is reported to contain the taxane binding site, yet mutations conferring paclitaxel resistance occur with equal frequency in both α and β-tubulin and both groups of mutants exhibit similar properties (Minotti et al. 1991; Hari et al. 2003; Yang et al. 2005). Many paclitaxel-resistant mutants require the presence of the drug for cell division and, therefore, must clearly retain the ability to bind the drug. Also, paclitaxel-resistant mutants frequently exhibit increased sensitivity to drugs such as colchicine and vinblastine that bind to distinctly different sites. Instead of altered drug binding, we favor a mechanism in which paclitaxel binding alters the conformation of both the M-loop and the short loop connecting H6 and H7 of β-tubulin in such a way as to facilitate and stabilize protein-protein interactions important in the formation of MTs. Recent fitting of the crystal structure of tubulin to a lower resolution map of the MT is consistent with the participation of the H6-H7 loop in both longitudinal and lateral contacts (Amos and Lowe, 1999). Because the mutations appear to destabilize MT assembly in the absence of any drug a direct effect on protein-protein interactions appears the more likely possibility.

This hypothesis is also supported by the recent observations of Xiao et al. (2006) who suggest that paclitaxel-binding may act to increase the energy associated with lateral and longitudinal interactions between tubulin dimers in the MT. Therefore changes within these surfaces would mitigate the action of energetic contributions of paclitaxel binding. Additionally, many of the mutations discussed here are not located within drug binding pockets at all, yet still confer drug resistance onto the cells in which they are found. In general we can consider that cells resistant to MT stabilizing drugs, such as the taxanes or epothilones, will produce less stable MTs in response. This is demonstrated by decreased drug induced MT stability and often a requirement on the drug for growth. Conversely, cells that become resistant to MT destabilizing drugs such as colchicine or vinblastine will produce MTs with increased stability. We must, however, be cautious when examining the data available in the literature, as much of the experimental results have been obtained through *in vivo* screening and do not give us specific information about paclitaxel binding to β tubulin. We believe that the key to developing new drugs that target tubulin and disrupt MT dynamics is through a better understanding of MT dynamics at the molecular level and the role mutations and isotype differences have on the dynamic properties of tubulin and the MT.

While it is clear that a substantial amount of work has to be done on obtaining specific binding data for drug binding to β-tubulin isoforms and mutants described here, the presence of minor variations within the structure of β-tubulin may provide us with a starting point for the development of novel drugs, or in the derivatization of existing drugs in order to gain in increased specificity or effect. This type of approach may also allow us to develop secondary treatments for cancer cell lines that have developed drug resistance as a result of standard chemotherapy treatments, due to mutations or altered expression levels. We feel that the most significant mechanism leading to drug resistance, as a result of both isotype expression and acquired mutations has to do with interactions within the MT structure itself and less to do with altered drug binding. This observation provides us with a novel set of targets with which to begin designing drugs that target these “hot spots” in an effort to counter the effects that β-tubulin isotype expression or mutations have on the development of drug resistance in these cells. We hope that the analysis presented here will provide deeper insights and perhaps a new direction for the future development of drugs that target crucial regions within the MT. It is our ultimate goal to develop a new generation of drugs that will target variant tubulins and thereby help reduce the adverse side effects associated with their use.

## Figures and Tables

**Table 1.** Observed β tubulin mutations in mammalian cell lines as a result of acquired drug resistance. The columns representing drug interactions indicate residues in which any atom is within 6 Å of any atom within the bound drug molecule from structural files 1JFF, 1SA0 and 1Z2B for Paclitaxel (PTX), Colchicine (CN) and Vinblastine (VLB) respectively.

**Mutation**	**Cell Type**	**Reference**	**Phenotype**	**Drug Interactions PTX CN VLB**
D45Y	CHO	Barlow 2002, Hari 2003, Wang 2004	Increased MT stabilization Growth dependent on Colchicine	
V60A,F	CHO	Wang 2004, Yang 2005	Decreased MT stabilization Taxol resistant Cis-acting suppressor of D45Y	
S172A	Human	Poruchynski 2004	Increased MT stabilization Hemiasterlin Colchicine and Vinca resistance.	*
P173A	CHO	He 2001	Decreased MT stabilization Epothilone A resistant.	*
D197N	Human	Gokmen 2005	Increased MT stabilization 2-Methoxyestradiol and Vinca resistant. No effect on Taxol or Colchicine phenotype	
C211F	CHO	Hari 2004	Increased MT stability Colchicine and vinca resistance	*
L215*	CHO	Gonzales 1999, Barlow 2002, Wang 2006	Decreased MT stabilization Taxol resistance. Not implicated in drug binding	*
L215V	CHO	Wang 2006	Normal Phenotype	*
L215I	CHO	Wang 2006	Decreased MT stabilization Taxol sensitivity, but not Epothilone Sensitive	*
L217R	CHO	Gonzales 1999	Decreased MT stabilization Taxol resistant. Not implicated in drug binding	*
D224N	CHO	Hari 2003	Increased MT stability Colchicine and vinca resistance	*
L228F,R	CHO	Gonzales 1999	Decreased MT stabilization Taxol resistance. Not implicated in drug binding	*
A231T	Human	Verrills 2003	Decreased MT stabilization Taxol resistant, hypersensitive to destabilizing compounds	*
S234N	CHO	Hari 2003	Increased MT stability Colchicine and vinca resistance	*
L240I	Human	Kavallaris	Increased MT stabilization Vinca and Colchicine resistant	*
F270V	Human	Giannakakou 1997	Decreased MT stabilization Taxol resistance	*
T274I	Human	Giannakakou 2000	Decreased MT stabilization Epothilone resistant reduced sensitivity to Taxol may affect drug binding	*
R282N	Human	Giannakakou 2000	Decreased MT stabilization Epothilone resistant reduced sensitivity to Taxol may affect drug binding	*
Q292H,E	CHO Human	He 2001, Verrills 2003, Wang 2004, Yang 2005	Decreased MT stabilization Taxol resistant Cis-acting suppressor of D45Y	
R306C	Human	Hasegawa 2002	Not known how this confers PTX resistance only a genetic study	
K350N	Human	Hari 2003, Gokmen 2005	Increased MT stabilization 2-Methoxyestradiol and vinca resistant. No effect on Taxol or Colchicine phenotype	*
A364T	Human	Giannakakou 1997	Decreased MT stabilization PTX resistance	*
Y442C	CHO	He 2001	Decreased MT stabilization Epothilone A resistant	

**Table 2.** Hot spots correlating between β-tubulin isotype sequences and β-tubulin mutations associated with acquired drug resistance. All residues identified as being nonconserved among the Human β-Tubulin isotypes were compared with those identified in Table 1. The isotypes in which the change occurs are identified with the differing residues in parentheses.

**Residue**	**Mutant**	**Mutant Phenotype**	**β-tubulin Isotype Expression**
45	D45Y	CN resistant	βI(D) βII–VIII(E)
172	S172A	CN resistant	βI–VI,VIII(S) βVII(L)β
231	A231T	PTX resistant	βI–V,VII,VIII(A) βVI(L)
364	A364T	PTX resistant	βI,VII(V) βIII,V(S) βII,IV,VI,VIII(A)
